# Advances in the treatment of relapsed/refractory chronic lymphocytic leukemia

**DOI:** 10.1007/s00277-017-2982-1

**Published:** 2017-04-07

**Authors:** C. Shustik, I. Bence-Bruckler, R. Delage, C. J. Owen, C. L. Toze, S. Coutre

**Affiliations:** 1 McGill University Health Centre, Montreal, Quebec Canada; 2 Department of Hematology, The Ottawa Hospital, Ottawa, ON Canada; 3 Department of Hematology, CHU de Québec-Université Laval, Quebec City, Quebec, Canada; 4 Division of Hematology and Hematological Malignancies, University of Calgary, Calgary, AB Canada; 5 Leukemia/BMT Program of BC, Division of Hematology, Vancouver General Hospital, BC Cancer Agency and The University of British Columbia, Vancouver, BC Canada; 6 Division of Hematology, Stanford University School of Medicine, Stanford, CA USA

**Keywords:** CLL, HSCT, Ibrutinib, Idelalisib, Venetoclax

## Abstract

Treatment of chronic lymphocytic leukemia (CLL) has advanced with the introduction of chemoimmunotherapy (CIT) agents that have improved the outcomes of frontline therapy. However, most treated patients will relapse and require subsequent therapy. This review focuses on recent advances in the treatment of relapsed or refractory CLL. Until recently, treatment options for relapsed CLL were of limited efficacy. Retreatment with fludarabine, cyclophosphamide, and rituximab (FCR) was recommended for patients with a durable response to first-line FCR, although acquired genetic aberrations, impaired marrow reserve, and comorbidities often made this suboptimal therapy for many patients. New options include two agents targeting B cell receptor (BCR) signaling pathways (ibrutinib and idelalisib) and a B cell lymphoma-2 (BCL-2) inhibitor (venetoclax). Allogeneic hematopoietic stem cell transplantation (HSCT) remains a potentially curative option for younger patients with a suitable donor.

## Introduction

Recent progress in the treatment of chronic lymphocytic leukemia (CLL) has been dramatic with the introduction of several novel agents. The addition of an anti-CD20 monoclonal antibody to frontline chemotherapy (chemoimmunotherapy [CIT]) results in improved progression-free survival (PFS) and overall survival (OS) compared to chemotherapy alone [1, 2]. However, even with excellent responses to frontline therapy, most patients will relapse with the need for subsequent treatment. The focus of this review will be on recent advances in the treatment of relapsed or refractory CLL.

Chronic lymphocytic leukemia is the most common lymphoproliferative disorder in adults, with an age-adjusted incidence of 4.8 to 5.0 per 100,000 person-years [3, 4] and a median age at diagnosis of 72 years [5]. Staging systems using clinical (adenopathy/organomegaly) and hematologic (anemia and thrombocytopenia) parameters remain useful in stratifying patients in this disease, which has a variable clinical course and a survival that may range from 18 months to more than 20 years [5, 6].

Although distinguished by a diagnostic immunophenotype, CLL has been characterized as a heterogeneous disease by genetic and molecular studies. These have identified useful prognostic and predictive variables correlating with the tempo of disease progression and survival, as well as response to therapy. Cytogenetic testing by fluorescence in situ hybridization (FISH) can detect several recurrent genetic aberrations in CLL with the most frequent abnormalities at diagnosis being 13q deletion (55% of patients), which is associated with a favorable prognosis; 11q deletion (18%) and 17p deletion (7% in previously untreated patients and about 30% in relapsed/refractory patients), both associated with a more rapidly progressive course and shorter survival; and trisomy 12 (16%), associated with an intermediate prognosis [7]. DNA studies of immunoglobulin genes in CLL have also defined two subsets of CLL with mutated or unmutated immunoglobulin heavy-chain variable (*IGHV*) regions based on sequence homology with germ line *IGHV* genes. Patients with unmutated *IGHV* have a shorter time to first treatment and PFS following CIT than patients with mutated *IGHV*. Zeta chain-associated protein kinase 70 kD (ZAP-70) impacts B cell receptor (BCR) signaling, proliferation, and migration and is predominantly expressed in the unmutated genotype, although it is not a reliable surrogate for unmutated *IGHV* status. Genomic studies have identified mutations involving notch homolog 1 (NOTCH1) and splicing factor 3b, subunit 1 (SF3B1), which also appear to predict a shorter time to treatment failure and reduced OS [8]. Telomere length has been shown to be a robust independent predictor of CLL outcomes, including OS and Richter’s transformation (transformation into a more aggressive large B cell lymphoma) [9]. An International Prognostic Index for CLL (CLL-IPI) incorporating five independent prognostic features (*TP53* status, age, clinical stage, *IGHV* mutational status, and β2-microglobulin level) has recently been developed to allow more targeted CLL patient management in clinical practice and clinical trials [10]. Using a weighted grading of these factors, it identifies the following four risk groups with significantly different OS at 5 years: low (93.2%), intermediate (79.3%), high (63.3%), and very high (23.3%) risk.

## Currently used therapies

Treatment of CLL is often deferred in asymptomatic, early-stage patients and initiated in the presence of signs or symptoms outlined by the International Workshop on CLL (IWCLL) criteria [11]. The choice of frontline CLL therapy is influenced by patient age and fitness. Aside from Eastern Cooperative Oncology Group (ECOG) performance status, an approach to formal evaluation of the latter has been the Cumulative Illness Rating Scale (CIRS), which rates comorbidities that may affect tolerability and toxicity of different regimens. With a wider range of therapeutic options now available, categorization of patients based on age (≥ or <65, 70, or 75 years), creatinine clearance (< or ≥70 mL/min), and fitness assessment has become important in the choice of therapy. Improvement in PFS and OS are common goals of therapy, but the risk/benefit ratio of different regimens may be distinct for different risk groups. In younger patients who are better able to tolerate more myelosuppressive regimens, a more intensive approach may be justified, whereas the primary objective in an elderly population may be quality of life with less focus on OS. In chemoimmunotherapy trials, complete response (by IWCLL criteria [11]) and achievement of minimal residual disease (MRD) status (<10^−4^ CLL cells detected by multiparameter flow cytometry) are correlated with PFS and OS prolongation. Although MRD negativity may be a clinically important endpoint in potentially curative strategies and in the design of maintenance therapy trials, MRD analysis is not routine in the current standard of care and remains investigational.

Randomized trials have established the combination of fludarabine, cyclophosphamide, and rituximab (FCR) as the frontline standard of care in a younger population with few comorbidities [1, 12]. A 10-year follow-up of 300 patients treated with FCR at MD Anderson Cancer Center indicated a sustained PFS in a subset of patients, with 42 patients experiencing no relapses beyond 10.4 years [13]. The best results were noted in patients lacking poor-risk FISH aberrations and with mutated *IGHV*. Older age (≥70 years) was associated with a lower rate of complete remission (CR; 51 vs. 76% in younger patients), and 54% of older patients did not complete the planned 6 cycles of therapy due to persistent cytopenias [14]. Based on a phase 2 study of bendamustine plus rituximab (BR) [15], the German CLL group conducted a phase 3 study comparing FCR to BR in previously untreated fit patients (CIRS score ≤6, creatinine clearance ≥70 mL/min) [16]. Although treatment with FCR was superior to BR overall for PFS (median 55.2 vs. 41.7 months; *p* = 0.0003, hazard ratio [HR] 1.643), no difference in overall survival was observed with a median follow-up of 37.1 months. Furthermore, hematologic toxicity and infectious complications were more pronounced with FCR in patients >65 years, and with concerns about protracted immunosuppression with FCR, the combination of bendamustine plus rituximab can be considered an acceptable treatment option for fit patients in this age group. A phase 3 trial in untreated CLL patients with a CIRS score >6 or a creatinine clearance of 30 to 60 mL/min randomized 781 patients with a median age of 73 years to treatment with chlorambucil (CLB), chlorambucil with rituximab, or chlorambucil with obinutuzumab, a humanized glycoengineered type 2 anti-CD20 MoAb. The latter combination resulted in improved OS compared to CLB and superior PFS and CR rate compared to CLB-rituximab [17] and currently defines the standard of care for this population of patients. Similarly, a phase 3 study comparing the combination of ofatumumab, an anti-CD20 that binds to a different epitope, and chlorambucil to chlorambucil alone confirmed the benefit of the combination in improving PFS in this population (22.4 vs. 13.1 months; *p* < 0.001) [18].

The decision to initiate therapy for relapsed CLL is based on the same considerations as for frontline treatment, although observation (i.e., “watch and wait”) in patients with slowly progressive lymphocytosis as the only disease manifestation may require closer monitoring than in untreated patients. Until the recent development of kinase inhibitors targeting B cell signaling pathways, treatment options in this population were of limited efficacy. The benefit of adding rituximab to FC in previously treated patients was shown in the REACH trial comparing FCR to FC with improved PFS in the CIT arm (median 30.6 vs. 20.6 months) [2]. Comparable results were observed in the COMPLEMENT 2 trial with the addition of ofatumumab to FC (median 28.9 vs. 18.8 months) [19]. These combinations might be considered for appropriate patients with limited prior therapies, and retreatment with FCR may be effective in patients with a durable response to frontline FCR (progression-free interval exceeding 24–36 months); however, bone marrow suppression is frequent and the duration of a second response is predictably shorter. Further complicating treatment in this setting is the observation of del (17p) and *TP53* mutation (by sequence analysis) in ~30% of relapsed patients post-FCR [20, 21], which predicts poor response to retreatment with purine nucleosides and alkylating agents. Impaired marrow reserve resulting from previous chemotherapy and additional comorbidities as a consequence of progression of disease and advancing age must also be considered in this setting.

In the minority of patients who are young and fit enough to be eligible, allogeneic hematopoietic stem cell transplantation (allo HSCT), harnessing a “graft vs. leukemia” effect, offers the best chance of cure.

### Allogeneic stem cell transplantation

For selected patients with high-risk CLL and adequate organ function as well as a suitable donor, allo HSCT may be the best option for prolonged survival and possible cure. The potential for long-term disease-free progression (OS 41–65% at 4–5 years [22–27]; see Table 1) must be balanced against the significant risk of chronic graft-versus-host disease (cGVHD; 44–65%) with associated morbidity and the risk of treatment-related mortality (TRM). This risk/benefit analysis is based on factors related to disease, patient, and donor [28]. Chronic lymphocytic leukemia with poor initial response to a purine analog-based regimen (<PR or relapse within 12 months from response) or progression within 24 to 36 months of CIT (FCR, BR, or other anti-CD20-based regimen) identifies high-risk patients [29]. However, the most recent American Society for Blood and Marrow Transplantation (ASBMT) guidelines no longer recommend considering these patients for allograft evaluation in the absence of high-risk FISH mutations (17p deletion, *TP53* mutation, or 11q deletion) [30]. Instead, novel agent therapy is proposed for these patients representing a change from previous European Blood and Marrow Transplant guidelines. Patients relapsing who have evidence of clonal evolution and/or complex karyotype, or with del (11q) with suboptimal response or del (17p), should be evaluated for transplant [31]. Novel agents are recommended first in this setting, but emerging data suggest shorter durations of response in patients with del (17p) or complex karyotypes and limited salvage options after failure of novel agents such that allo HSCT should be entertained ideally prior to loss of disease control by novel agents.

**Table 1:** Allogeneic stem cell transplantation in relapsed CLL

	Dreger 2010 [22]	Khouri 2011 [23]	Sorror 2010 [24]	Brown 2012 [25]	Toze 2012 [26]	Hebenstreit 2014 [27]
*N*	90	86	136	76	49	50
Median age (years)	53	58	56	55	54	58
PFS	42% at 4 years	36% at 5 years	32% at 5 years	43% at 5 years	49% at 5 years	63% at 4 years
OS	65% at 4 years	51% at 5 years	41% at 5 years	63% at 5 years	55% at 5+ years	51% at 4 years
Relapse	40% at 4 years	39% at 3 years	36% at 5 years	40% at 5 years	16% at 5 years and 22% at 15 years	37% at 4 years
Extensive cGVHD	55% at 2 years	56% at 5 years	51%	65% at 2 years	57% at 5 years	44%
NRM	23% at 4 years	17% at 1 year	32% at 5 years	16% at 5 years	36% at 10 years	30% at 4 years

Predictors for PFS and OS were CR and absence of bulky disease (lymph nodes >5 cm)

*cGVHD* chronic graft-versus-host disease, *NRM* nonrelapse mortality

Patient selection for allo HSCT is also based on careful consideration of comorbidities that affect TRM and availability of a suitable donor (ideally either a matched sibling or fully matched unrelated donor). The use of reduced-intensity conditioning (RIC) regimens with lower TRM may extend the age eligibility for suitable patients. The integration of novel agents into the pre- or post-transplant setting of allo HSCT is also under investigation. Published data have suggested that ibrutinib may be used safely both pre- and post-allo HSCT [32]. While phase 3 data are lacking, the benefits of allo HSCT in high-risk CLL patients in prolonging PFS have been shown in several studies (Table 1). However, these studies were completed before the use of novel agents, and it is not known if these results will hold in the current setting.

The ASBMT guidelines recommend allo HSCT (a) for standard-risk CLL in the absence of response or if there is evidence of disease progression after BCR inhibitors, (b) for high-risk CLL after failing two lines of therapy and showing an objective response to BCR inhibitors or to a clinical trial or for patients who fail to show an objective response or progress after BCR inhibitors and receive B cell lymphoma-2 (BCL-2) inhibitors regardless of whether an objective response is achieved, and (c) for Richter’s transformation upon demonstration of an objective response to anthracycline-based chemotherapy [30]. A RIC regimen is recommended as appropriate.

## Newly approved agents

### Ibrutinib

The molecule ibrutinib was developed as an oral irreversible inhibitor of BTK, an intracytoplasmic enzyme in the BCR signaling pathway that is required for BCR activation of integrins and other molecules. Congenital mutations or absence of BTK in humans leads to profound deficiency of B lymphocytes due to arrest in B cell development with consequent agammaglobulinemia. In CLL, BCR signaling is aberrantly activated, promoting B cell proliferation and survival as well as modulating migration and homing of malignant cells. The anti-tumor activity of ibrutinib (Table 2) results from disruption of BCR signaling as well as targeting of toll-like receptor signaling and adhesion and migration pathways.

**Table 2:** Trials involving new agents

Drug	Phase	Number	Prior lines of therapy (median)	ORR	CR	PFS	*p* Value
Ibrutinib [20]	1b/2	85	4	71%	2%	75% at 26 months	n/a
Ibrutinib vs. ofatumumab [37]	3	391	3	42.6 vs. 4.1%	2 vs. 1%	Not reached vs. 8.1 months	*p* < 0.001 for ORR
Ibrutinib [38]	1b/2	101^a^	4	90%	7%	69% at 30 months	n/a
Idelalisib + rituximab vs. placebo + rituximab [52]	3	220	Anti-CD20-based or ≥2 previous	81 vs. 13%	0%	Not reached vs. 5.5 months	*p* < 0.001 for ORR
Venetoclax [60]	1	56	4	84%	21% CR/Cr_i_	n/a	n/a
Venetoclax [61]	1	116	3	79%	20%	25 months	n/a
Venetoclax [63]	2	107	2	79%	8%	Not reached at 12.1 months	n/a
Venetoclax + rituximab [62]	1	49	2	84%	41% CR/Cr_i_	n/a	n/a

^a^Relapsed/refractory CLL patients only

An initial phase 1b/2 clinical trial in relapsed/refractory CLL [20] studied the safety, efficacy, and pharmacokinetics of ibrutinib. Eighty-five patients with a median of 4 (1–12) prior therapies, almost all exposed to a purine nucleoside and rituximab, were treated with ibrutinib at a dose of 420 mg (*n* = 51) or 840 mg (*n* = 34). Deletion 17p was present in 33% and del (11q) in 36%. The overall response rate (ORR) was 71% at both dose levels, and responses were independent of adverse cytogenetics. At 26 months, PFS for the entire cohort was 75% and OS 83%. The responses were predominantly partial with the observation of an early transient increase in lymphocytes, with frequent persistent peripheral lymphocytosis, despite regression of adenopathy and splenomegaly and improvement in other hematologic values. This phenomenon of an early rise in lymphocyte count has been attributed to dislodging of CLL cells from nodal compartments into the circulation. The recognition that persistent or increased lymphocytosis is not indicative of treatment failure with these agents has necessitated a revision of response criteria with the addition of partial response with lymphocytosis (PR_L_) [33]. Based on the results of these early phase studies, ibrutinib received FDA-accelerated approval in relapsed CLL in 2014 [34], then was accepted as a breakthrough drug for CLL with 17p deletion the same year [35]. This study established the 420-mg daily dose for subsequent trials with identical BTK occupancy at 96 to 99% for both the 420- and 840-mg doses [36].

The efficacy of ibrutinib was confirmed in the RESONATE^™^ trial, a phase 3 comparison of ibrutinib to ofatumumab in patients with relapsed or refractory CLL with PFS as the primary endpoint [37]. Eligibility criteria included at least one prior therapy and ineligibility for purine analog treatment due to comorbidities, age over 70 years, presence of del (17p), or short duration of response after CIT. In this multicenter study, 391 patients were randomly allocated to receive ibrutinib 420 mg daily until disease progression (*n* = 195) or ofatumumab at an initial dose of 300 mg, followed by 2000 mg weekly for 7 weeks, then every 4 weeks for 16 weeks (*n* = 196). The baseline characteristics were well balanced with a median of 3 (1–12) prior therapies in the ibrutinib group and 2 (1–13) in the ofatumumab group; del (17p) and del (11q) were each detected in about 30% of patients in both arms. The study was terminated after a pre-planned interim analysis demonstrated markedly improved outcomes for the ibrutinib arm. With a median follow-up of 9.4 months, median PFS in the ofatumumab arm was 8.1 months and had not been reached in the ibrutinib arm with a HR for progression or death in the ibrutinib arm of 0.22 (*p* < 0.001). A crossover design permitted patients progressing on ofatumumab to receive ibrutinib once the primary endpoint was reached, and at the time of analysis, 57 patients had crossed over to ibrutinib. Nonetheless, an OS advantage for the ibrutinib therapy was observed in both uncensored and censored for crossover groups (HR for death in ibrutinib arm 0.39 and 0.43, respectively, at 12 months; OS 90% in ibrutinib group and 81% in ofatumumab group). Improvement in PFS was observed across all subgroups regardless of age, clinical stage, *IGHV* mutation status, or presence of del (17p).

An update of the initial phase 1b/2 trial reported 3-year follow-up of 31 treatment-naïve (TN) patients and 101 patients with relapsed/refractory CLL treated with single-agent ibrutinib [38]. At a median of 30 and 23 months on study for TN and relapsed/refractory patients, 81 and 53%, respectively, remained on drug. Response quality improved with time; with extended follow-up, 94% of patients who achieved PR_L_ converted to CR or PR. Discontinuation due to disease progression was only 3% in the TN group but 21% in the relapsed/refractory group, whereas discontinuation due to drug intolerance was similar in both groups (10 and 12%, respectively). The estimated PFS at 30 months was 96 and 69% for the two groups but only 48% in patients with del (17p).

The toxicities observed with ibrutinib are modest, with the majority of reported adverse events (AEs) being grade 1–2. The most frequent nonhematologic AEs occurring in at least 20% of patients were diarrhea, bleeding, fatigue, pyrexia, and nausea. In the RESONATE study, AEs of grade 3 or higher in the ibrutinib arm included atrial fibrillation (AF) in 3%, although subsequent reports noted increasing AF prevalence with additional time on ibrutinib [39]. Another study found that about 6% of all newly diagnosed CLL patients had a history of AF; in those without such a history, the background CLL population incidence of AF was about 1% per year [40]. A recent systematic review and meta-analysis found that the pooled relative risk of AF associated with ibrutinib as compared to the comparator in randomized trials was 3.5 to 3.9, depending on the model used. The pooled rate of AF among ibrutinib recipients from all trials examined was 3.3 per 100 person-years [41]. Arrhythmic symptoms or new-onset dyspnea in patients receiving ibrutinib should be evaluated clinically, with electrocardiography if appropriate. Ibrutinib therapy should be withheld in patients with new-onset or worsening grade 3 or 4 toxicities and reinitiated at the starting dose once symptoms have resolved [42]. In the RESONATE, RESONATE-2, and HELIOS trials, most patients with AF were able to continue ibrutinib treatment and did not discontinue due to AF [39, 43, 44].

Bleeding-related AEs, most commonly petechiae or ecchymoses, have also been reported with ibrutinib (44% with ibrutinib vs. 12% with ofatumumab in RESONATE), but major hemorrhage (grade 3 or higher or requiring red cell transfusion or hospitalization) occurred in only two patients in the ibrutinib group and three in the ofatumumab group. A study of single-agent ibrutinib in CLL found that the cumulative incidence of grade ≤2 bleeding-related AEs plateaued by 6 months, suggesting that the risk of bleeding decreases with continued therapy [45]. Ibrutinib should be withheld for at least 3 to 7 days pre- and post-surgery depending on the type of surgery and the risk of bleeding, and vitamin K antagonists should not be administered concomitantly. If therapeutic anti-coagulation is required, consider temporarily withholding ibrutinib until stable anti-coagulation is achieved [42].

As impaired humoral immunity and increased infection risk resulting from panhypogammaglobulinemia are characteristic of advanced CLL, the effect of BTK inhibition on normal B cell function in CLL may have clinical relevance. A study of 86 patients with previously untreated or relapsed/refractory CLL receiving ibrutinib for at least 12 months [46] found a progressive decline in serum immunoglobulin G (IgG) levels, while immunoglobulin A (IgA) levels increased with treatment. In patients with a ≥50% increase in IgA level, the infection rate was decreased, suggesting partial immune recovery with ibrutinib therapy. Patients taking ibrutinib in RESONATE also experienced increased IgA levels, as well as sustained improvements in hemoglobin, platelet levels, and absolute neutrophil count (ANC) compared with patients taking ofatumumab [47]. Another study found low rates of treatment-emergent autoimmune cytopenias (AICs) with ibrutinib treatment, and 19 of 22 patients receiving corticosteroids for autoimmune hemolytic anemia at the start of ibrutinib therapy were able to discontinue them with resolution of the hemolytic process [48].

### Idelalisib

Idelalisib (Table 2) is an orally bioavailable inhibitor of the delta isoform of PI3K, the predominant PI3K isoform in B cells. PI3K has limited expression in other hematopoietic cells, and thus, PI3K inhibition acts as a targeted B cell therapy. As an inhibitor of PI3K signaling downstream from the BCR in CLL cells, this drug also interrupts BCR signaling pathways. However, idelalisib may also disrupt the protective effect of the CLL microenvironment [49] by interfering with chemokine networks, including CXCR4, CD40, and CD49d effects on multiple signaling pathways [50]. It was approved by the FDA in 2014 for the treatment of relapsed CLL in combination with rituximab [51].

In phase 1 studies, idelalisib was investigated as a single agent and in combination with many other chemoimmunotherapeutic agents in relapsed or refractory CLL patients. The clinical activity and acceptable toxicity led to a pivotal phase 3 randomized trial of idelalisib plus rituximab vs. rituximab plus placebo [52]. Patients were eligible if they had progressed within 24 months of their last treatment (which must have included an anti-CD20-based therapy or at least two prior cytotoxic regimens) and were not candidates for cytotoxic drugs due to impaired marrow reserve as a consequence of prior myelosuppressive therapy, or a creatinine clearance <60 mL/min, or a CIRS score >6. Of the patients, 222 were allocated to treatment with rituximab 375 mg/m^2^ as an initial dose, followed by 500 mg/m^2^ every 2 weeks for four doses then every 4 weeks for three doses (for a total of eight infusions) in combination with either idelalisib 150 mg or placebo twice daily. Patients (median age 71 years) were stratified by *IGHV* mutation status and the presence of del (17p) or *TP53* mutation (present in 40%). Baseline characteristics, including hematologic values, CIRS scores, and number and type of prior therapies, were well balanced. At 24 weeks, 93% of patients in the rituximab-idelalisib group were progression-free compared to 46% in the rituximab-placebo arm, and the study was stopped at this pre-specified point. Median PFS in the rituximab-placebo arm was 5.5 months and had not been reached in the idelalisib with rituximab cohort (HR for progression or death in the idelalisib arm, 0.15; 95% confidence interval 0.08 to 0.28; *p* < 0.001). This clinical benefit for the combination was observed in all pre-specified subgroups including high-risk patients with del (17p) and/or *TP53* mutation. Updated results of this study [53] reported a median PFS of 16.6 months in the latter group and 20.3 months in patients without either abnormality.

In this study, the most frequently observed grade 3 or higher adverse events attributed to idelalisib were diarrhea (5%) and increases in hepatic transaminases (8%) [53]. Adverse events of any grade included neutropenia (60%), transaminase elevation (40%), anemia (29%), thrombocytopenia (19%), bleeding (14%), pneumonia (10%), rash (10%), and pneumonitis (6%). Diarrhea/colitis of any grade occurred in 21% of patients on idelalisib and was managed by drug interruption, corticosteroids, and symptom management. The incidence of diarrhea/colitis is underrepresented in the early publications due to limited time on therapy for most patients at the time of analysis. Later reports with longer follow-up report a higher incidence of diarrhea/colitis, an adverse event that requires careful monitoring and rapid treatment [54]. The distinct late-onset diarrhea occurs with a median onset of more than 6 months of therapy and may be associated with colitis with lymphocytic infiltration on biopsy [55]. This adverse event requires drug interruption and typically requires steroid therapy for rapid resolution. Either prednisone or nonabsorbable corticosteroids (e.g., budesonide) are generally effective in ameliorating this condition. Once the diarrhea resolves, the patient can often be successfully rechallenged with idelalisib and the steroids tapered off. Hepatic enzyme elevations were reversible by withholding drug, and idelalisib could be restarted in most patients without recurrence. Pneumonitis has also been reported with idelalisib in other studies, including fatal cases, without identifiable pathogens and with no defined mechanism. A 2015 consensus paper summarizes experience in the management of these more concerning toxicities of idelalisib [56]. During safety monitoring of several trials of idelalisib in untreated CLL and relapsed low-grade lymphoma, an increased incidence of serious infection was observed and these trials were terminated. Health care professionals were advised of these results by the manufacturer (Gilead Sciences), and prophylaxis for *Pneumocystis carinii* and monitoring for cytomegalovirus reactivation have consequently been mandated during idelalisib treatment [57].

### Venetoclax

Venetoclax (Table 2), a BH3 mimetic, is an orally administered small molecule that potently inhibits the anti-apoptotic BCL-2 protein with limited effect on BCLX_L_, a related anti-apoptotic protein important for platelet survival. Venetoclax induces rapid onset apoptosis of CLL cells, apparently independently of *TP53* function. Analysis of both in vivo and in vitro results from a phase 1 trial showed that the depth of clinical responses to venetoclax were also independent of chromosome 17p deletion, *TP53* mutation, and *TP53* function [58]. This agent has recently been approved by the FDA for the treatment of previously treated CLL with 17p deletion in the USA [59] and is currently being studied in phase 2 and 3 trials.

In an initial phase 1 trial [60], 56 patients with relapsed/refractory CLL or small lymphocytic lymphoma (SLL), including 17 with del (17p), were treated with single-agent venetoclax. The ORR was 84% (82% in del [17p]) including rates of 20% CR or CR with incomplete marrow recovery (Cr_i_). Tumor lysis syndrome (TLS) developed in five patients, leading to the suspension of the trial to reassess dosing of the drug. The trial was restarted with a weekly stepwise dosing schedule with no subsequent observed TLS.

Roberts reported the results of a phase 1 study of venetoclax in combination with rituximab in 49 patients with relapsed/refractory CLL [61]. A CR/Cr_i_ rate of 41% was observed, including MRD negativity in 65% (13/20) of these patients (49% [24/49] overall). These responses were sustained in six patients achieving CR/CR_i_ for up to 21 months after discontinuing treatment. The most frequent grade 3 or higher AEs were neutropenia (51%), thrombocytopenia (16%), and anemia (14%), and there was one death due to TLS.

The updated results of the initial phase 1 dose-escalation study of daily oral venetoclax, including an expansion cohort of 60 additional patients, have recently been reported [62]. In the dose-escalation phase, 56 patients with relapsed/refractory CLL or SLL received daily venetoclax in doses ranging from 150 to 1200 mg. In an expansion cohort of 60 additional patients, venetoclax was escalated in weekly stepwise increments to 400 mg daily. Patients enrolled in this study had received a median of three prior lines of therapy although none with prior ibrutinib or idelalisib. Patients with autoimmune cytopenias were excluded. Pooled ORRs of 71 to 79% were observed in subgroups with adverse prognostic features including fludarabine resistance, del (17p), and unmutated *IGHV*. Complete remissions were observed in 20% of the patients in both cohorts, and 5% had undetectable MRD by flow cytometry. With a median follow-up of 17 months in the cohort treated at 400 mg daily, the median PFS could not be reliably estimated, but the rate of PFS was estimated to be 66% at 15 months with the likelihood that the CR rate would increase with longer observation. In the expansion cohort following dose-escalation adjustments, no cases of clinical tumor lysis were observed.

A multicenter, phase 2, single-arm study examined venetoclax monotherapy (using a stepped-dose schedule) in relapsed or refractory del (17p) CLL [63]. At a median follow-up of 12.1 months, an ORR of 79% was achieved (85 of 107 patients), with a CR/Cr_i_ of 8%, a nodular PR of 8%, and a PR of 69%. MRD in peripheral blood was not detectable in 18 of 45 assessed patients. The most common grade 3–4 adverse events were neutropenia (40%), infection (20%), anemia (18%), and thrombocytopenia (15%). Serious adverse events occurred in 55% of patients, irrespective of their relationship to treatment, with pyrexia, autoimmune hemolytic anemia, pneumonia, and febrile neutropenia seen most commonly. Laboratory TLS was reported in five patients during the ramp-up period (four within the first 2 days of treatment and one at week 3) but resolved without clinical sequelae.

Despite the lack of clinical TLS after the institution of the slow stepwise increase in dose of venetoclax, it remains important to monitor for laboratory abnormalities indicating TLS, particularly in patients considered at higher risk because of a significantly elevated blood lymphocyte count (>25 Gi/L) or those with bulky adenopathy (>5 cm). In appropriate cases, hospitalization for dose escalations of venetoclax is required.

## Discussion

### Patient selection for specific agents

Despite the improvement in response rates and disease control with frontline chemoimmunotherapy in CLL, disease relapse remains the norm. Although a subset of patients with mutated *IGHV* may experience long PFS after CIT with FCR, the majority of patients will eventually require subsequent therapy. With the broader availability of newer agents, the challenge for the clinician is selection and sequencing of these drugs.

Retreatment with FCR may be considered for suitable patients experiencing an initial PFS exceeding 24 to 36 months; however, impaired marrow reserve following this treatment and the emergence of a del (17p) clone may limit the efficacy of this regimen. In addition, there is significant concern about the increased risk of myelodysplasia with repeated exposure to fludarabine. In a minority of younger patients, allo HSCT should be considered a potentially curative approach if a human leukocyte antigen (HLA)-matched donor is available, but for most patients, transplantation will not be feasible due to age and/or comorbidities and many patients will achieve good results with novel agents in this setting.

While disease relapse characterized by slowly progressive lymphocytosis may not require immediate reinstitution of therapy, subsequent treatment decisions will be guided by the same factors determining initial therapy, including patient age and concurrent comorbidities, as well as marrow reserve, which may be impaired as a result of prior treatment. Repeat cytogenetic assessment should be performed since the presence of del (17p) is critical to treatment decisions, and the frequency of this event increases with subsequent relapses. Current options for treatment with novel drugs have been reviewed above. In the absence of evidence from randomized trials directly comparing the agents under discussion, preferences may be determined by patient characteristics. Ibrutinib, idelalisib, and venetoclax are all active in relapsed CLL with del (17p). With respect to depth of response beyond CR, MRD negativity has been associated with ibrutinib in combination (18% in the HELIOS trial in combination with BR) [64], but rarely with ibrutinib monotherapy. Venetoclax has been associated with MRD responses when given as monotherapy (17%) [63]. The impact of achieving this degree of response on OS in relapsed CLL remains to be established; ibrutinib has demonstrated conclusive OS benefit in randomized trials without achieving MRD negativity. The ease of administration of oral, once-daily ibrutinib vs. the concomitant requirement for 8 cycles of intravenous rituximab with oral, twice-daily idelalisib may be a consideration in favor of ibrutinib for some patients, as may the option for dose reduction in patients with comorbidities (although dose reductions for this reason are based on physician preferences rather than trial data). Conversely, the need for anti-coagulation therapy or a prior history of atrial fibrillation may favor idelalisib, depending on clinician preference. An indirect comparison of ibrutinib monotherapy and idelalisib plus ofatumumab [65] suggested a longer PFS and fewer discontinuations with ibrutinib, although a head-to-head trial is required for a true comparison. In appropriate patients (those who have achieved a lengthy first remission after CIT), retreatment with CIT remains a reasonable option and has the advantage of a short duration of therapy and a subsequent treatment-free interval.

### Resistance, progression, and sequencing

Disease progression occurring in patients after prolonged treatment with ibrutinib has been associated with poor prognosis and short survival (median 17.6 months after CLL progression and 3.5 months if Richter’s transformation had occurred) [66]. However, the patients included in this analysis were from early clinical trials with ibrutinib and had largely exhausted standard treatment options when they entered the trials (median of three prior therapies). In an analysis of RESONATE and RESONATE-2 patients, 23 patients who had discontinued first- or second-line ibrutinib had not yet reached a median OS, compared with a median OS of 7–9 months in 34 patients who discontinued third-line ibrutinib or beyond [67]. Of the 31 previously untreated patients who received ibrutinib as initial therapy in the phase 1 trial [68], only one has been reported with subsequent ibrutinib failure. Richter’s transformation has been reported as an early complication in the course of therapy with ibrutinib, but typically in previously treated patients with adverse cytogenetic features, including complex karyotype and *MYC* abnormalities on FISH; these patients may have had early Richter’s at study entry. Studies in patients developing resistance to ibrutinib have identified point mutations in the BTK binding site C481S, resulting in loss of BTK inhibition, and in the immediate downstream kinase PLCγ2 [69]. A retrospective analysis of 123 CLL patients who discontinued ibrutinib- or idelalisib-based therapy found that many patients who discontinued these therapies due to toxicity or progression responded to other therapies (40% PR + PR_L_ to nonkinase inhibitors and 60–67% to other kinase inhibitor therapy) [70]. Preliminary results from an ongoing phase 2 trial also suggest that venetoclax monotherapy is active in CLL patients relapsing after idelalisib or ibrutinib [71].

Lenalidomide, an immunomodulatory drug with potent in vitro activity in CLL, remains under investigation in CLL, but one randomized study comparing it to chlorambucil was terminated early due to excess mortality in the lenalidomide arm [72]. Acalabrutinib, a second-generation BTK inhibitor that does not irreversibly target alternative kinases, has been investigated in a phase 1/2 study [73]. A phase 3 study comparing this drug to ibrutinib in high-risk patients with relapsed CLL has been initiated. Clinical trials of other agents including XPO1 inhibitors (e.g., selinexor), Syk inhibitors (fostamatinib and entospletinib), new BTK inhibitors (BGB-311), and new PI3K inhibitors (buparlisib, duvelisib, and TGR-1202) are also in progress. The dramatic responses observed with CAR-T cell therapy in small numbers of advanced, refractory CLL patients suggest the possibility of effective immunotherapeutic strategies in the future.

### Combination therapy

Ibrutinib and idelalisib have each been combined with BR in phase 3 clinical trials comparing the three-drug combination with BR alone. In both cases, the addition of the new agent resulted in significant improvements in outcome [74, 75]. Two-year results for the HELIOS trial, which studied the combination of ibrutinib with BR, found that over a median follow-up of 25.4 months, the triple combination was superior to BR alone in PFS (not reached vs. 14.2 months), 2-year PFS (74.8 vs. 20.9%), CR/Cr_i_ (33.9 vs. 7.2%), best ORR at any time point (87.2 vs. 66.1%), and MRD-negative response (18.0 vs. 4.8% in the intent-to-treat population). Median OS remained unreached in both treatment arms and there were no new safety findings [64].

The combination of idelalisib and ofatumumab was compared with ofatumumab alone in an open-label phase 3 study in 261 patients with relapsed CLL [76] (Table 3). The combination demonstrated superior median PFS (16.4 vs. 8.0 months; *p* < 0.0001) and ORR (75.3 vs. 18.4%; *p* < 0.0001).

**Table 3:** Summary of phase 3 trial results in relapsed/refractory CLL

Trial	Agents	Design	Number	Median age	Median PFS (months)	Median follow-up (months)	MRD
REACH [2]	FCR vs. FC	Open label	552	62.5	30.6 vs. 20.6 months (*p* < 0.001)	25	13 vs. 12%
RESONATE [37]	Ibrutinib vs. ofatumumab	Open label	391	67	Not reached vs. 8.1 months	9.4	n/a
Furman 2014 [52]	Idelalisib + rituximab vs. rituximab	Double-blind	220	71	Not reached vs. 5.5 months	3.8 and 2.9^a^	n/a
HELIOS [74]	Ibrutinib + BR vs. BR	Double-blind	578	63.5	Not reached vs. 13.3 (*p* < 0.0001)	17	18 vs. 5% (*p* = 0.0011)
Zelenetz 2015 [75]	Idelalisib + BR vs. BR	Double-blind	416	58% <65 years	23 vs. 11 (*p* < 0.0001)	12	n/a
COMPLEMENT 2 [19]	Ofatumumab + FC vs. FC	Open label	365	61	28.9 vs. 18.8 (*p* = 0.0032)	34	21 vs. 8% (*p* = 0.0006)
Jones 2016 [76]	Idelalisib + ofatumumab vs. ofatumumab	Open label	261	67	16.4 vs. 8.0 (*p* < 0.0001)	12.3	n/a

^a^Time receiving study drug

The combination of idelalisib and entospletinib was tested in a phase 2 trial and produced an ORR of 60% in relapsed CLL patients with a median treatment exposure of 10 weeks. However, the study was terminated early due to treatment-emergent pneumonitis in 18% of patients (12 cases, 11 of which were severe), resulting in two fatalities [77].

The combination of ibrutinib and FCR was studied as first-line therapy in young, fit CLL patients in a phase 2 trial. Early results in 17 restaged patients showed an ORR of 100% after a median of 7.7 months of therapy, 8 patients (47%) with CR or CR_L_, all of whom were MRD-negative, and 9 (53%) with PR [78].

A number of other combination regimens involving agents discussed above are in ongoing clinical trials in CLL patients. Regimens under investigation include ibrutinib plus obinutuzumab, ibrutinib plus lenalidomide, ibrutinib plus lenalidomide plus rituximab, ibrutinib plus selinexor, and venetoclax plus ibrutinib plus obinutuzumab. The combination of venetoclax and sunitinib may also be worth investigating, based on the results of an in vitro study that found that sunitinib may overcome venetoclax resistance in some patients by downregulating BCL-xl, Mcl-1, and A1 in CLL cells [79].
